# Evaluation of Anesthesiology Residents’ Supervision Skills: A Tool to Assess Transition Towards Independent Practice

**DOI:** 10.7759/cureus.4137

**Published:** 2019-02-26

**Authors:** Efrain Riveros Perez, Enoe Jimenez, Nianlan Yang, Alexander Rocuts

**Affiliations:** 1 Anesthesiology, The Medical College of Georgia, Augusta University, Augusta, USA

**Keywords:** clinical supervision, anesthesiology residency, educational intervention, competence

## Abstract

Problem

Anesthesiologists are often expected to supervise residents, nurse anesthetists, and anesthesiologist assistants in their practice. Development of a supervisory skill set is important during anesthesiology training and has a potential impact on the quality of patient care. During anesthesiology residency training, residents develop different competencies through direct supervision by a staff anesthesiologist. However, there is significant variability among anesthesia residency programs in the United States in terms of the opportunity residents have to supervise other anesthesia providers. The supervisory competency is not routinely evaluated during residency training.

Intervention

This study aimed at evaluating an educational seminar to foster the competency of supervision in anesthesiology. The 90-minute seminar included a live lecture and a series of workshops. The lecture had a duration of 45 minutes followed by three workshops of 15 minutes each. The workshops consisted of different simulated case scenarios with the participation of actors and a manikin as a patient. A debriefing session took place after the scenarios. Every resident included in the study participated in the workshops. The workshops were aligned with the learning objectives of the educational strategy.

Context

The study included 12 junior anesthesiology residents supervised by 24 senior residents during simulated clinical encounters. Quality of supervision, using the nine-item Quality of Supervision Questionnaire validated by De Oliveira Filho, and self-perception were evaluated before and after the educational intervention consisting of a face-to-face seminar and individual workshops administered during each encounter.

Impact

There was a significant difference between the overall means among senior residents for the quality of supervision measured by a nine-item quality of supervision questionnaire before and after the educational intervention program (3.11 ± 0.29 vs 3.96 ± 0.17, p < 0.01). There was no significant difference between the overall means for the self-perception of the senior residents before and after the intervention program (3.51 ± 0.54 vs. 3.48 ± 0.20).

Lessons learned

A bimodal educational intervention combining face-to-face seminars and workshops is effective to improve the quality of supervision in senior residents; however, it does not change the self-perception of the supervisory process. Addition of this type of educational intervention to the anesthesiology residency curriculum would add to the development of the supervisory competency and warrants further research in clinical situations.

## Introduction

During anesthesiology training, residents develop different competencies through one-on-one interaction with a staff anesthesiologist. However, there is little opportunity during the training period to acquire the skills necessary to supervise other anesthesia providers. Supervision is indeed one of the major responsibilities of faculty anesthesiologists. The term "supervision" encompasses “all clinical oversight functions directed toward assuring the quality of clinical care whenever the anesthesiologist is not the sole anesthesia care provider” [1]. Effective supervision entails specific skills that are different from those associated with general competencies [2]. It also serves the dual purpose of graduate medical education and billing compliance [3]. Effective supervision focuses on quality and safety of both the patient and the trainee in the context of clinical care [4]; it should include gradual delegation of responsibilities, as well as constant evaluation and sharing of clinical judgment. Despite the crucial role that supervision plays in resident training, it has been one of the least researched areas in education [5].

The characteristics of an effective supervisor have been studied in different educational fields. There seems to be agreement on the importance of aspects, such as an opportunity to perform procedures, direct involvement in patient care, and constructive feedback [6]. Other features identified by trainees as important qualities in a supervisor include the supervisors’ teaching and interpersonal communication skills [7]. Regarding psychological traits, an effective supervisor is supportive, flexible, focused, practical, respectful, and is interested in supervision [8]. Finally, serving as a role model and providing feedback have been important aspects identified for effective supervision [9].

In the context of the complexity of interactions between the supervisor and trainees in the clinical setting, training for supervisors is necessary [10] (and probably essential in anesthesiology) since residents abruptly transition from being supervised trainees to become supervisor attending anesthesiologists. In clinical fields, such as nursing and psychiatry, some authors have advocated for the selection of professionals to take supervisor training courses [11]. These courses are structured based on the needs of the supervisor, as well as for the purposes of supervision [12]. The practice model in anesthesia is founded on physician supervision and medical direction rather than direct physician-only care, making training in supervision a valuable tool in preparation to enter clinical practice as a staff anesthesiologist.

Supervision of anesthesiology residents is part of the faculty activities contemplated by the Accreditation Council for Graduate Medical Education (ACGME) and is to be evaluated on a regular basis by the supervised residents [13]. De Oliveira Filho et al. validated a nine-item supervision scale with application to overall departmental and rotation settings [1, 14]. The De Oliveira Filho instrument [15] can also assess the evaluation of individual anesthesiologist’s supervising performance. Each question from 1 to 9 in the scale evaluates a different dimension, including feedback, availability, stimulus to patient-based learning, professionalism, presence, peri-anesthesia planning, safety, interpersonal skills, and opportunity/autonomy. This measurement instrument has been found to have internal consistency, face and content validities, and unidimensional factor structure. However, one of its limitations is the possibility of some degree of halo error, which may affect the ranking of evaluated individuals depending on the evaluating resident [16].

Supervision of other anesthesia providers by an anesthesiologist is a unique skill that plays an important role in both quality patient care and professional development of anesthesia trainees. Acquisition of this skill must be part of the anesthesiology training program, and it would be appropriate to start its evaluation before the resident graduates. This study evaluated the effectiveness of an educational intervention consisting of a virtual module course on the residents’ skills as supervisors, using the nine-item supervision scale validated by De Oliveira Filho.

## Materials and methods

Participants and methods

After approval by the Augusta University Institutional Review Board, 36 anesthesiology residents were invited to participate in the study. Twelve residents from each postgraduate clinical anesthesia years (CA-1, CA-2, and CA-3) were enrolled in the study. Clinical encounters in the operating room occurred with the presence of a junior resident, a senior resident acting as a supervisor, and an attending anesthesiologist responsible for the room. The supervision ratio of the junior: senior resident was 1:1. The attending anesthesiologist oversaw the process and was readily available for questions from the senior resident. The junior resident notified any changes during the case to both the attending anesthesiologist and the senior resident. The residents (junior and senior) were not paired beforehand, and the same junior resident could evaluate different senior residents on different encounters. The same attending anesthesiologist could monitor more than one room where the encounters took place. The overarching supervision of the attending anesthesiologist was not standardized, and their supervision was not evaluated in this study. There was no standardization for patient surgical case complexity. On the other hand, junior residents received input from the senior resident before the attending gave feedback in order to reduce the influence of the attending feedback on the interaction between the junior and senior residents. The study was divided into two phases. In the first phase, the residents completed questionnaires assessing the quality of the supervision received by junior residents (CA-1) from senior residents (CA-2 and CA-3) during clinical encounters and self-perception as supervisors by senior residents. The encounters took place during clinical cases under the overarching supervision of an attending anesthesiologist.

Educational intervention

Table 1 details the aims, content, and pedagogical design of the educational intervention. A panel comprising staff anesthesiologists and pedagogical advisors provided input in the design of this seminar of effective supervision. The seminar had a duration of 90 minutes, was presented by the investigators (ERP, AR), and included a live lecture and a series of workshops. The lecture had a duration of 45 minutes, followed by three workshops of 15 minutes. The workshops, consisting of different simulated case scenarios, were presented by the investigators with the participation of actors and a manikin as the patient. A debriefing session took place after the scenarios. Every resident included in the study participated in the workshops. The workshops were aligned with the learning objectives of the educational strategy (Table 1).

**Table 1:** Educational Intervention PowerPoint® (Microsoft® Corp., Redmond, WA

Aims, content, and pedagogical design of intervention in the effective supervision for anesthesiology residents
Intervention aims:
To improve senior anesthesiology residents’ skills, knowledge, and attitudes toward supervision
To raise awareness of the importance of supervision in the practice of anesthesiology
To identify opportunities and methods to provide effective feedback within a supervisory relationship.
Educational intervention content:
The anesthesiology supervisor role
Models of clinical supervision
Process of supervision
Ethical and legal aspects related to supervision
Supervision and relationship supervisor/supervised individual
Feedback models
The resident with difficulties
Diagnostic analysis
Pilot survey
Pedagogical strategy
Lectures (45 minutes – All participants)
Prepared by a group of faculty anesthesiologists with experience in education
Review of supervisory competence and relevance
Journey from unconscious incompetence (the residents is oblivious to his/her lack of supervision skills) to unconscious competence (supervision skills have been acquired and are applied automatically in practice).
Principles of effective supervision
Feedback models
Workshops – Clinical scenarios (45 minutes per participant)
Clinical scenario in the simulation lab
Debriefing and feedback
Resource book
Goals and objectives
PowerPoint^®^ presentation slides
Suggested further reading

Our educational intervention was planned by a collaborative effort of pedagogical advisors from Augusta University and our research team. We constructed the aims, content, and format (Table 1). We identified four lecture topics: standards of supervision, relevance to the anesthesiologist, feedback, and effective supervision. The purpose of the intervention was to improve senior residents’ skills, knowledge, and attitudes toward supervision. The intervention was designed to include the following components and formats: (1) 45 minutes of lecture, including a case discussion, and (2) three workshops of 30-minute duration each per resident, consisting of patient case simulation scenarios with a manikin and actors representing different roles in the operating rooms. During the workshops, the anesthesiology resident played the role of the attending anesthesiologist. The workshop supervisor addressed critical supervision and feedback issues during the scenario. After the simulation scenario, a debriefing session took place where the key messages presented during the lecture were emphasized. The resident was given the opportunity to assess his/her own performance during debriefing.

Measures

Tables 2-3 show the instruments used to measure different aspects related to supervision. Self-perception as a supervisor was evaluated before the educational intervention and after the first encounter with a junior resident. The De Oliveira Filho Nine-item Quality of Supervision Questionnaire [14] was completed by junior residents after the encounters with senior residents. Scores of this questionnaire evaluating the quality of supervision during encounters before and after the lecture and workshops were compared to assess the effectiveness of the intervention. The post-intervention encounters started after one week of the administration of the intervention. Of note, the nine-item De Oliveira Filho questionnaire refers to the supervisor as an instructor. The term was clarified to the participating residents to maintain the original form of the validated instrument.

**Table 2:** Quality of Faculty Supervision Questionnaire Evaluation of each item with the scale 1 = Strongly disagree, 2 = Agree, 3 = Neutral, 4 = Disagree, 5 = Strongly agree. Items adapted to anesthesia residents from De Oliveira Filho et al. [14].

Quality of Supervision
The instructors discuss with me the perioperative management of patients prior to starting an anesthetic procedure and accept my suggestions when appropriate.
The instructors are present during critical moments of the anesthetic procedure (induction, complications, emergence).
The instructors give me opportunities to perform procedures appropriate to my training level and stimulate my independence.
The instructors are promptly available to solve problems and help me with procedures.
The instructors demonstrate theoretical knowledge, proficiency at procedures, ethical behavior, and interest/compassion/respect for patients.
The instructors use real clinical scenarios to stimulate my clinical reasoning, critical thinking, and theoretical learning.
The instructors treat me respectfully, creating and maintaining a pleasant, non-threatening environment during clinical activities.
The instructors teach and require the implementation of safety measures during the perioperative period (e.g., anesthesia machine checkout, universal precautions, prevention of medication errors, etc.).
The instructors provide timely, formative feedback showing me ways to improve my performance.

**Table 3:** Supervisor Self-evaluation Evaluation of each item with the scale 1 = Strongly disagree, 2 = Agree, 3 = Neutral, 4 = Disagree, 5 = Strongly agree. Items adapted to anesthesia residents from Saarikoski et al. [17].

Self-evaluation
My attitude towards supervising other residents was positive all the time.
I felt comfortable supervising other residents.
I discussed goals and objectives for the case with the supervised resident.
The relationship with the supervised resident was good during the case.
I am able to assess the performance of the supervised resident to establish a plan of learning.
I feel confident with my abilities to supervise.
I feel I don’t have enough authority to direct the actions of the supervised resident.
I feel that supervision of more than one operating room could be overwhelming to me.
I felt I needed the backup of my staff anesthesiologist to make decisions about the case while I was supervising a resident.
I think I am able to supervise all types of activities during a case.
I feel afraid of not having enough skills to take over the case should the supervisee fails a procedure on the first attempt.
I feel I have enough knowledge to supervise others.
I tolerate supervisee’s inadequacies/problems during the learning process.
I tolerate criticism from my supervisee.

Quality of supervision

The questionnaire evaluating the quality of supervision consisted of nine items, each evaluating a single dimension of supervision. Each question was graded on a 4-point rating scale (never = 1, rarely = 2, frequently = 3, and always = 4). The supervision score equaled the mean of the scores for each item (1, 26).

Study methodology

First, we administered the Nine-item Quality of Supervision Scale by de Oliveira de Filho (Table 2) to junior residents (CA-1) supervised by a senior anesthesia resident (CA-2, CA-3). The encounters took place during clinical cases under the overarching supervision of an attending anesthesiologist. The clinical encounters were scheduled from the day before the scheduled procedure. The junior resident evaluated the patient and then discussed the case by phone with the attending anesthesiologist and the senior resident. The day of the surgery, the anesthetic plan was developed as discussed, simulating a regular anesthetic case. During this encounter, the junior resident received input from the senior resident before he/she met with the attending anesthesiologist. Additionally, self-perception as a supervisor was evaluated in senior residents by an 8-item questionnaire that forms part of a validated instrument to assess the learning environment and supervision [17] (Table 3). After the administration of the aforementioned questionnaires, the senior residents attended the 90-minute seminar (Table 1). After the seminar, the second set of supervision encounters with participating junior and senior residents took place. Finally, junior residents were asked to complete the Nine-item Quality of Supervision Questionnaire, and senior residents were asked to complete the self-perception survey. Overall, 36 encounters occurred before the educational intervention and 36 encounters occurred after the intervention.

We recognize that the validity of our findings might be affected by variables, such as history, maturation, instrumentation, and testing. Since we had pre- and post-intervention analysis on one group of subjects, the history and maturation were potential threats and we tried to control it by the short duration of the study. We used standardized instruments to mitigate the instrumentation threat.

Statistical analysis

Descriptive statistics were used to interpret the responses of the supervisees and supervisors. Means (M), standard deviations (SD), and 95% confidence intervals (CI) were calculated for the perceived supervisory quality. Student's independent t-test was used to identify differences between the responses before and after the intervention. Statistical significance was defined as a P-value < 0.05.

## Results

**Figure 1:**
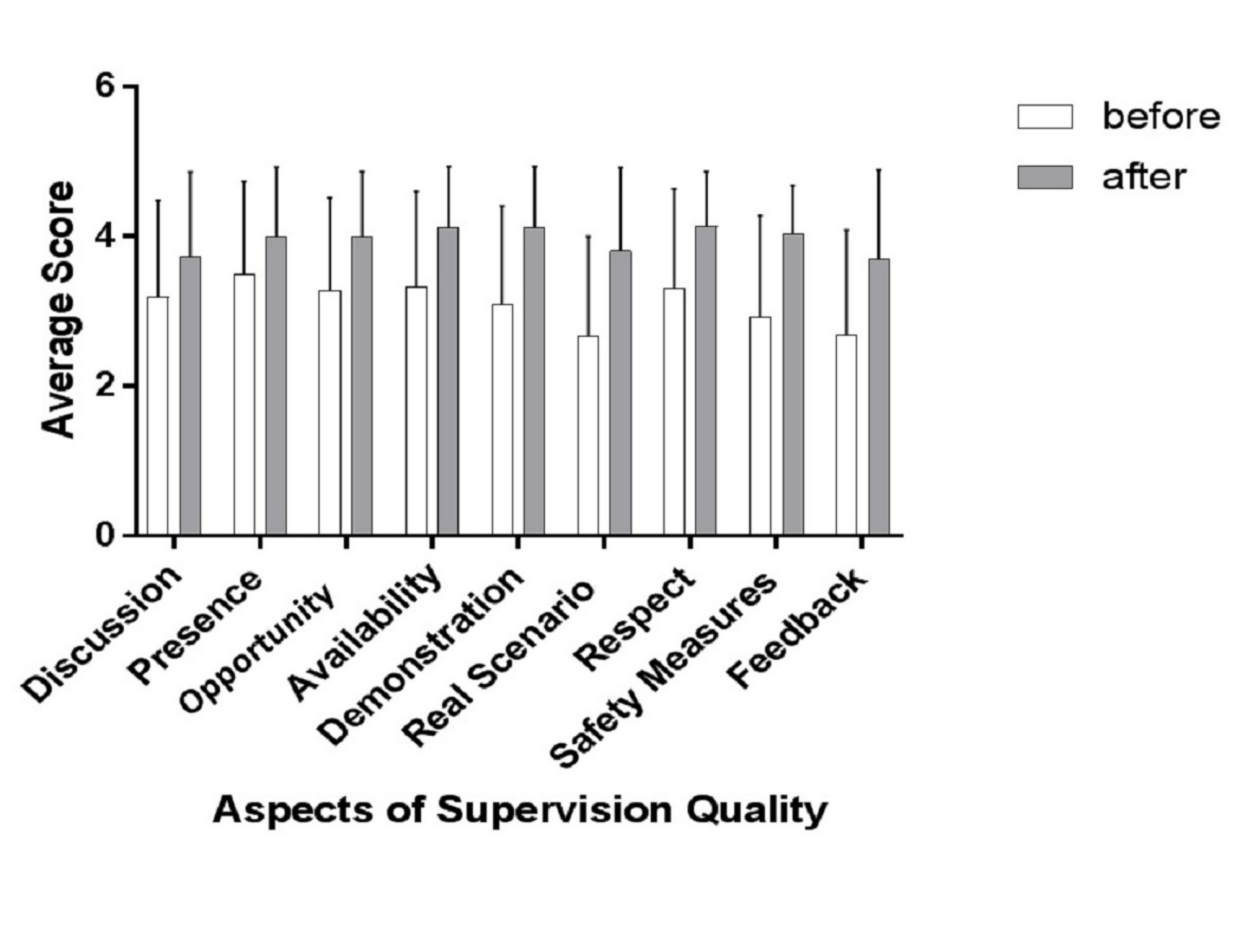
Comparison of different aspects of quality of supervision before and after the educational intervention

This study showed that a comprehensive educational intervention consisting of a face-to-face lecture strategy, supplemented by clinical scenario workshops, improved the quality of supervision provided by senior anesthesiology residents. Table 4 shows an overview of the quality of supervision as perceived by junior residents. The results are statistically and clinically significant. There was a significant difference between the overall means for the quality of supervision as perceived by junior residents before and after the educational intervention program (3.11 ± 0.29 vs 3.96 ± 0.17, p < 0.01). All aspects included in the quality of the supervision questionnaire showed significant improvement, except for the items regarding “instructor discussed with me prior to starting a procedure” and “instructors were present during critical moments”. Figure 1 shows the improvement in individual aspects of the supervision quality.

**Table 4:** Overview of the Quality of Supervision as Perceived by the Junior Residents Using a Scale of 1 to 5* * 1 = Strongly disagree, 2 = Disagree, 3 = Neutral, 4 = Agree, 5 = Strongly agree. SD: standard deviation; CI: confidence interval

Supervisory Quality	Before			After			P value
	mean	(SD)	95% CI	mean	(SD)	95% CI	
1. The instructor discussed with me the perioperative management of patients prior to starting an anesthetic procedure and accepted my suggestions when appropriate.	3.19	(1.28)	2.77 - 3.61	3.72	(1.14)	3.35 - 4.09	0.07
2. The instructors were present during critical moments of the anesthetic procedure (induction, complications, emergence).	3.50	(1.23)	3.1 - 3.9	4.00	(0.93)	3.7 - 4.3	0.06
3. The instructor gave me opportunities to perform procedures appropriate to my training level and stimulated my independence.	3.28	(1.23)	2.88 - 3.68	4.00	(0.86)	3.72 - 4.28	<0.01
4. The instructors were promptly available to solve problems and help me with procedures.	3.33	(1.26)	2.92 - 3.74	4.11	(0.82)	3.84 - 4.38	<0.005
5. The instructors demonstrated theoretical knowledge, proficiency at procedures, ethical behavior, and interest/compassion/respect for patients.	3.08	(1.32)	2.65 - 3.51	4.11	(0.82)	3.84 - 4.38	<0.005
6. The instructors used real clinical scenarios to stimulate my clinical reasoning, critical thinking, and theoretical learning.	2.67	(1.33)	2.24 - 3.1	3.81	(1.12)	3.44 - 4.18	<0.005
7. The instructors treated me respectfully, creating and maintaining a pleasant, non-threatening environment during clinical activities	3.31	(1.33)	2.88 - 3.74	4.14	(0.72)	3.90 - 4.38	<0.005
8. The instructors teach and require the implementation of safety measures during the perioperative period	2.92	(1.36)	2.48 - 3.36	4.03	(0.65)	3.82 - 4.24	<0.005
9. The instructors provided timely, formative feedback showing me ways to improve my performance	2.69	(1.39)	3.14 - 2.23	3.69	(1.19)	4.07 - 3.30	<0.005

**Figure 2:**
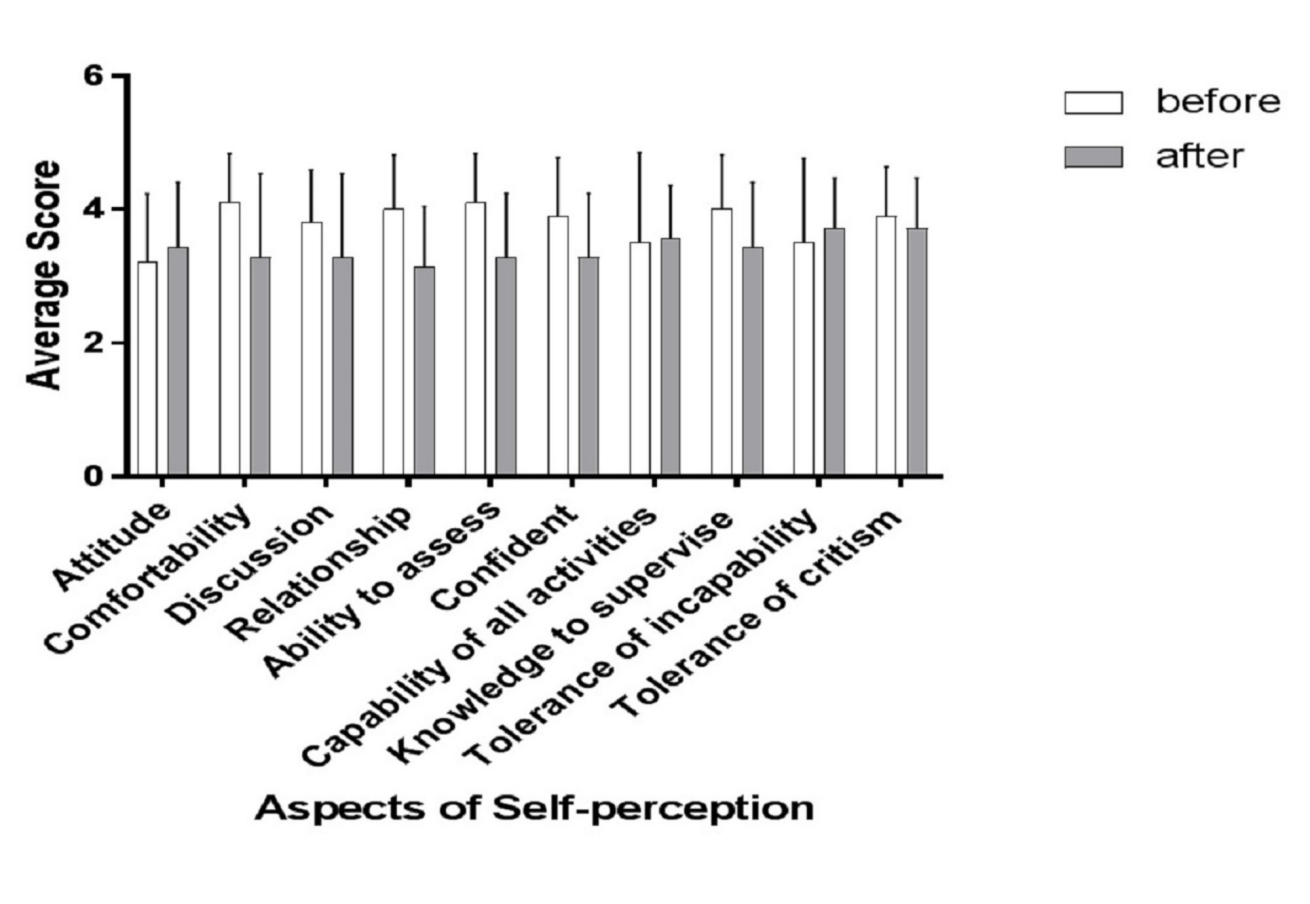
Changes in supervision self-perception before and after the educational intervention

**Figure 3:**
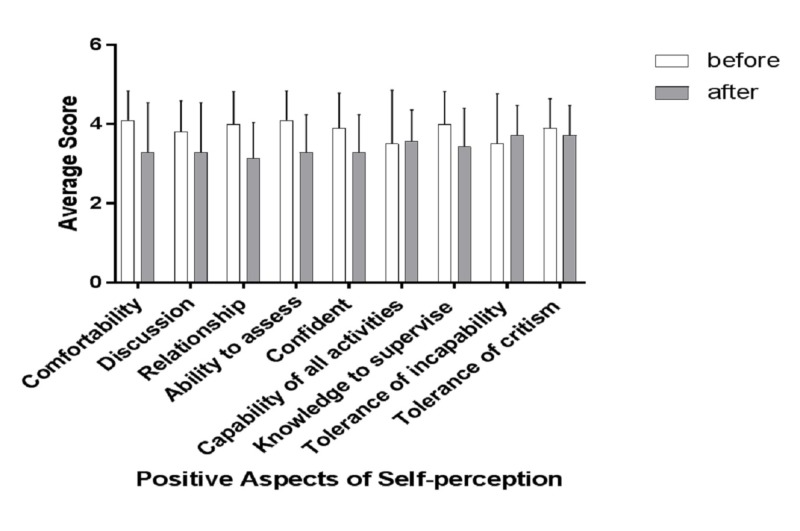
Changes in positive aspects of self-perception before and after the educational intervention

**Figure 4:**
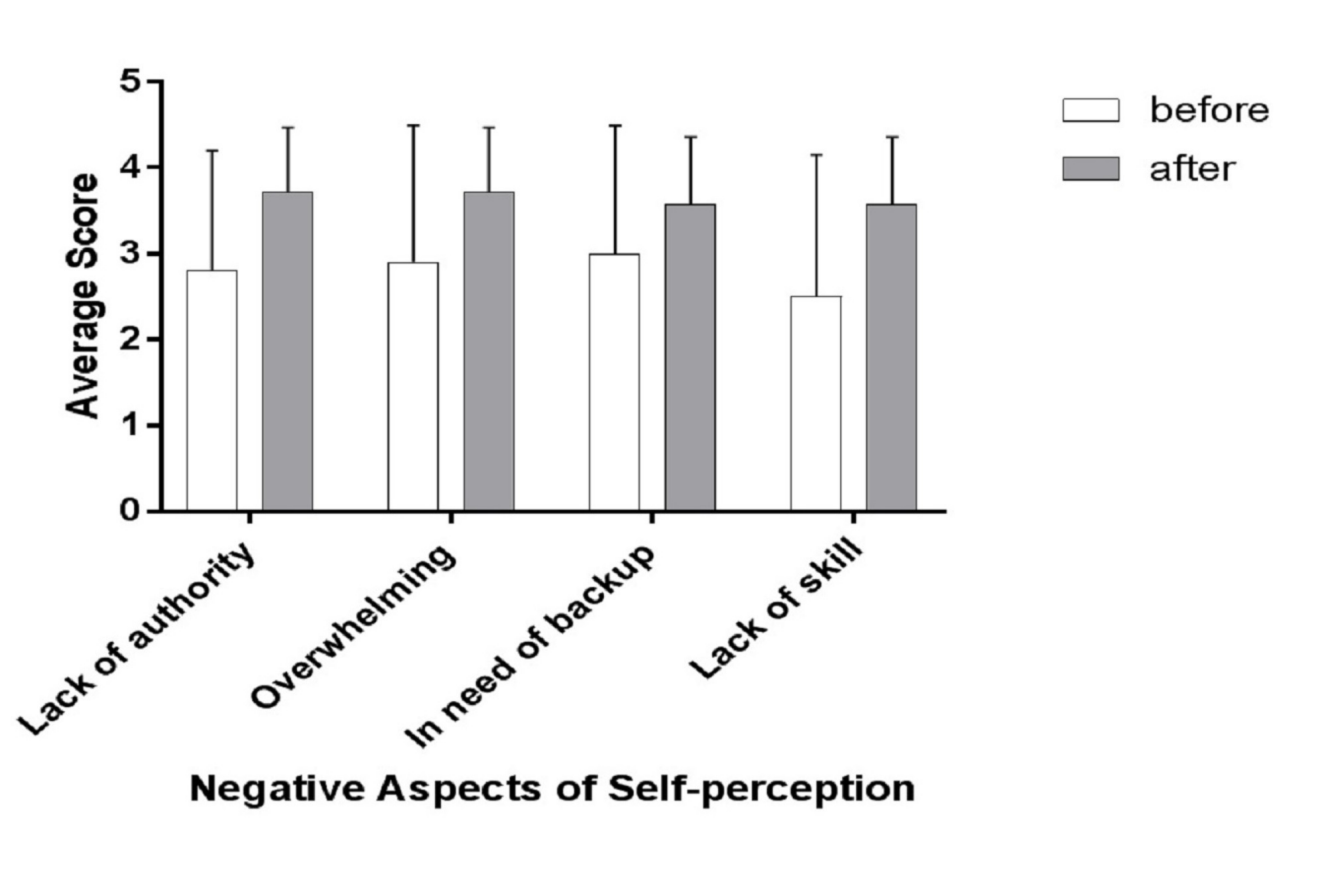
Changes in negative aspects of self-perception before and after the educational intervention

**Figure 5:**
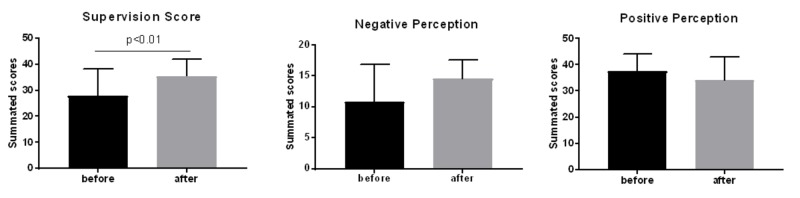
Composite scores for supervision; positive and negative perception of supervision

Table 5 shows an overview of the self-perception as a supervisor from the perspective of senior residents before and after the intervention. The post hoc power analysis results are consistent with the pre hoc analysis (Table 6). There was no significant difference between the overall means for the self-perception of the senior residents before and after the intervention program (3.51 ± 0.54 vs. 3.48 ± 0.20). However, the overall means for the positive aspects (items 1 - 10) decreased after the program (3.8 ± 0.30 vs. 3.41 ± 0.2, p = 0.01), and the overall means for the negative aspects (items 11 - 14) increased after the program (2.80 ± 0.22 vs. 3.64 ± 0.08, p = 0.01). None of the individual aspects showed any significant changes before and after the program (Figures 2-4). The composite analysis of the scale shows the summated scores for the supervision scores, as well as positive and negative perception (Figure 5). P-values were determined as part of the post hoc power analysis.

**Table 5:** Overview of the Supervision Self-perception by the Senior Residents Using a Scale of 1 to 5* * 1 = strongly disagree, 2 = disagree, 3 = neutral, 4 = agree, 5 = strongly agree CI: confidence interval; NS: not significant; SD: standard deviation

	Before			After			
	Mean	(SD)	95% CI	Mean	(SD)	95% CI	P-value
1. My attitude towards supervising other residents was positive all the time.	3.20	1.03	2.56 - 3.84	3.43	0.98	2.7 - 4.16	NS
2. I felt comfortable supervising other residents.	4.10	0.74	3.64 - 4.56	3.29	1.25	2.36 - 4.22	NS
3. I discussed goals and objectives for the case with the supervised resident.	3.80	0.79	3.31 - 4.29	3.29	1.25	2.36 - 4.22	NS
4. The relationship with the supervised resident was good during the case.	4.00	0.82	3.49 - 4.51	3.14	0.90	2.47 - 3.81	NS
5. I am able to assess the performance of the supervised resident to establish a plan of learning.	4.10	0.74	3.64 - 4.56	3.29	0.95	2.59 - 3.99	NS
6. I feel confident with the abilities to supervise.	3.90	0.88	3.35 - 4.45	3.29	0.95	2.59 - 3.99	NS
7. I think I am able to supervise all types of activities during a case.	3.50	1.35	2.66 - 4.34	3.57	0.79	2.98 - 4.16	NS
8. I feel I have enough knowledge to supervise others.	4.00	0.82	3.49 - 4.51	3.43	0.98	2.7 - 4.16	NS
9. I tolerate supervisee's inadequacies/problems during the learning process.	3.50	1.27	2.71 - 4.29	3.71	0.76	3.15 - 4.27	NS
10. I tolerate criticism from my supervisee.	3.90	0.74	3.44 - 4.36	3.71	0.76	3.15 - 4.27	NS
11. I feel I don't have enough authority to direct the actions of the supervised resident.	2.80	1.40	1.93 - 3.67	3.71	0.76	3.15 - 4.27	NS
12. I feel that supervision of more than one operating room could be overwhelming to me.	2.90	1.60	1.91 - 3.89	3.71	0.76	3.15 - 4.27	NS
13. I felt I needed the backup of my staff anesthesiologist to make decisions about the case while I was supervising a resident.	3.00	1.49	2.08 - 3.92	3.57	0.79	2.98 - 4.16	NS
14. I feel afraid of not having enough skills to take over the case should the supervisee fail a procedure on the first attempt.	2.50	1.65	1.5 - 3.5	3.57	0.79	2.98 - 4.16	NS

**Table 6:** Summary of Summated Scores. Analysis of the Scale Composite with Post Hoc Power

	Before	After	P value	Actual power (post hoc)
Supervision score	27.92 ± 1.711, n = 36	35.53 ± 1.077, n = 36	< 0.01	100%
Positive perception	37.6 ± 2.067, n = 10	34.14 ± 3.327, n = 7	0.366	70%
Negative perception	10.9 ± 1.882, n = 10	14.57 ± 1.131, n = 7	0.156	90%

## Discussion

This study demonstrated the effectiveness of an educational intervention consisting of a combination of a face-to-face seminar with individual clinical scenario workshops on the quality of supervision in senior anesthesiology residents, although it did not significantly change the self-perception as a supervisor in this study group.

Enhanced clinical supervision is associated with improved clinical and educational outcomes. Faman et al. conducted a systematic review evaluating both educational and patient outcomes [18]. Their study showed that direct or indirect supervision of trainees in the operating room had either a positive effect or no effect on complication rates, mortality, and postoperative pain. It also showed that attending supervision had a positive effect on resident compliance with the quality of care guidelines. Snowdon et al. showed that effective supervision was associated with improved effectiveness of care [19]. Our study demonstrated that with adequate intervention, we could contribute to the development of strong supervisory competence. Our study showed the effectiveness of an intervention administered to training anesthesia providers regarding the quality of supervision. We expect that this type of intervention translates into better patient care. In our study, we considered CA-2 level residents as senior trainees. Although still far from graduation, a CA-2 resident has completed the majority of rotations in general operating rooms, which was the scenario for the encounters. On the other hand, since our ultimate goal is to improve the skills and knowledge necessary to conduct effective supervision, we deemed it appropriate to start our educational intervention as early as the second year of clinical anesthesia training.

Some studies have evaluated the quality of supervision in anesthesiologists overseeing professional activities of residents and nurse anesthetists. Different authors have recognized the need to train anesthesiologists as supervisors; however, to our knowledge, no study has evaluated the supervisory competence of anesthesia trainees. Adame et al. found that the implementation of 30-minute feedback training was associated with the increased provision of positive feedback [20]. The authors recommend feedback training for attending anesthesiologists. Anesthesiology residents perform emergency intubations and other invasive procedures in critical situations. Training of residents in technical skills may not be sufficient when those same residents have to supervise other anesthesia providers after graduation. Schmidt et al. demonstrated that quality supervision during emergency tracheal intubation led to a decreased rate of complications [21]. In addition, quality of supervision is an independent factor to an anesthesiologist’s clinical value and his/her contribution to patient care [22].

In our study, self-perception before and after the educational intervention did not change significantly; however, factors (such as level of comfort, relationship with the supervisee, ability to assess performance, level of confidence, knowledge to supervise, and tolerance to supervisee’s inadequacy) showed lower scores after the program. On the other hand, characteristics (such as perception of one's own authority, the perception of simultaneous supervision as being overwhelming, the perception of insufficient knowledge to supervise, and the perception of need of an attending backup) showed worse scores after the intervention. Overall, these findings show that in the process of development of the supervisory competence, the supervisor resident transitioned from a state of unconscious incompetence to one of conscious incompetence, which is reflected in poor self-perception. However, self-perception is multifactorial in nature, and it may be affected by variables, such as diverse as emotional status and interactions with patients and healthcare personnel. Interestingly, the parameter of self-perception did not exhibit a difference between years of training among senior residents. A commonly accepted education model states that a trainee progresses through a sequence of skill acquisition consisting of four steps: 1) unconscious incompetence, 2) conscious incompetence, 3) conscious competence, and 4) unconscious competence [23]. Our study shows that the residents are moving in the right direction and need additional interventions and reinforcement to complete the four steps of competence before graduation. It is possible that educational programs, such as the one we used in this study, achieve better results if implemented early in training. In addition, residents are used to having control over the management of their own patients. Delegating responsibility to someone else is a new experience that generates anxiety. A process to develop trust in the supervisee is a process that requires training during residency [24].

Balancing teaching and safe care is of the utmost importance in acute care settings [25]. This balance warrants the development of teaching abilities by the clinician and teaching interactions to encourage progressive autonomy in trainees [26]. Lifelong learning has been recommended to improve the quality and safety of patient care [27]. Our study is aligned with the concept of lifelong learning from the early stages of anesthesia training. We consider that developing the competence to supervise is a process that should start in a controlled safe educational environment, such as the period that a trainee spends doing a residency. After graduation, the future anesthesiologist will be able to use the critical concepts of supervision and feedback to engage into a continual learning process aimed at improving the ability to provide safe care by means of interaction with midlevel anesthesia providers and residents.

Limitations

Our study has limitations. We studied residents from a single residency program, which might not be representative of the whole population of anesthesiology trainees in the United States; however, supervision practices and ratios are homogeneous across the country, which makes our findings relevant in relation to anesthesiology training in general. The answers to the quality of the supervision questionnaire, provided by junior residents, may have been affected by interpersonal relationships with supervisors. The supervisors’ interpersonal affective regard is associated with higher performance appraisal ratings [28], whereas leniency of the rating of the anesthesia resident affects the reliability of the quality of the anesthesiologists’ quality of clinical supervision [29]. We tried to control this relational factor by making the process anonymous; yet, we recognize that it may still have affected the results. Anesthesiologists supervise residents, anesthesiologist assistants (AA), and certified registered nurse anesthetists (CRNA) in their daily practice. We only included supervision of residents. Although we acknowledge the importance of supervision of all groups of anesthesia providers, we explored the supervision of residents by residents to focus on the educational aspects of supervision that are unique in an academic setting. Our study evaluated one dimension that is relevant to training anesthesiology residents; however, we did not focus on the educational aspects inherent to the intervention. Future research is needed to assess the pedagogical aspects of the seminar we used. Finally, the level of maturity and autonomy differs significantly between CA-2 and CA-3 residents [30]. The latter group of residents is close in performance to the role of an attending anesthesiologist. However, we decided to include CA-2 residents as supervisors as part of an educational program to improve supervisory competence. Future research should focus on the evaluation of supervision directed towards other groups of anesthesia providers, such as nurse anesthetists. Follow-up studies should address the progression of residents from the state that we achieved (conscious incompetence to unconscious competence) in terms of supervision of anesthesia providers during residency training.

## Conclusions

The educational intervention consisting of lecture/case discussion and workshops in simulation scenarios was effective to improve the quality of supervision in senior residents but failed to change the self-perception of the supervisory process. Despite the overall positive results, aspects (such as discussion prior to anesthetic procedures and physical presence at critical times) were not affected by the educational intervention. Future research is necessary to extend the scope of our study to other populations of anesthesia providers, including nurse anesthetists and anesthesiologist assistants.
